# Caught in the middle with multiple displacement amplification: the myth of pooling for avoiding multiple displacement amplification bias in a metagenome

**DOI:** 10.1186/2049-2618-2-3

**Published:** 2014-01-30

**Authors:** Rachel Marine, Coleen McCarren, Vansay Vorrasane, Dan Nasko, Erin Crowgey, Shawn W Polson, K Eric Wommack

**Affiliations:** 1 Delaware Biotechnology Institute, University of Delaware, 15 Innovation Way, Newark, DE 19716, USA; 2 Washington College, 300 Washington Avenue, Chestertown, MD 21620, USA; 3 Delaware Technical Community College, 400 Stanton-Christiana Road, Newark, DE 19713, USA

**Keywords:** Metagenomics, Microbial ecology, Multiple displacement amplification, PacBio SMRT sequencing, DNA library construction

## Abstract

**Background:**

Shotgun metagenomics has become an important tool for investigating the ecology of microorganisms. Underlying these investigations is the assumption that metagenome sequence data accurately estimates the census of microbial populations. Multiple displacement amplification (MDA) of microbial community DNA is often used in cases where it is difficult to obtain enough DNA for sequencing; however, MDA can result in amplification biases that may impact subsequent estimates of population census from metagenome data. Some have posited that pooling replicate MDA reactions negates these biases and restores the accuracy of population analyses. This assumption has not been empirically tested.

**Results:**

Using mock viral communities, we examined the influence of pooling on population-scale analyses. In pooled and single reaction MDA treatments, sequence coverage of viral populations was highly variable and coverage patterns across viral genomes were nearly identical, indicating that initial priming biases were reproducible and that pooling did not alleviate biases. In contrast, control unamplified sequence libraries showed relatively even coverage across phage genomes.

**Conclusions:**

MDA should be avoided for metagenomic investigations that require quantitative estimates of microbial taxa and gene functional groups. While MDA is an indispensable technique in applications such as single-cell genomics, amplification biases cannot be overcome by combining replicate MDA reactions. Alternative library preparation techniques should be utilized for quantitative microbial ecology studies utilizing metagenomic sequencing approaches.

## Background

Metagenomics has revolutionized the field of microbial ecology, providing a culture-independent means of studying the structure and metabolic potential of a microbial community. Obtaining sufficient quantities of high-quality DNA for sequencing is a consistent technical challenge for many metagenomics studies, and is especially the case for studies of viral communities. To circumvent low DNA yields from environmental samples, several amplification methods have emerged, with each method having specific advantages and drawbacks. Linker amplified shotgun library (LASL) procedures require as little as 1 pg of DNA and minimize %GC content amplification bias (≤1.5-fold), but are low throughput [1]. Transposase-based protocols (e.g., Nextera, Illumina Corp., San Diego, CA, USA) [2] and linear amplification for deep sequencing (LADS) [3] protocols require slightly greater quantities of DNA (1 to 40 ng), with Nextera being better adapted for high-throughput library preparation, albeit with an acknowledged bias against higher %GC DNA content as compared to linker amplified metagenomes [4].

Multiple displacement amplification (MDA) has been one of the most commonly used means of amplifying environmental genomic DNA (gDNA), especially viral gDNA, prior to the construction of DNA fragment sequencing libraries [5]. This technique utilizes the phi29 DNA polymerase, and is capable of producing long fragments (12 kb average) under isothermal conditions [6]. While MDA provides an easy and effective means of amplifying minute quantities of DNA, biases associated with this technology, including chimera formation, preferential amplification of circular single stranded DNA (ssDNA) and non-uniform amplification of linear genomes, have been documented [7,8]. Furthermore, the ability to accurately estimate the frequency of individual populations from multiple displacement amplified environmental gDNA has been challenged in controlled experiments [9]. MDA-induced errors in population frequency estimates are believed to arise from preferential amplification of particular genomic regions during initial MDA priming events [10,11]. Several investigators have proposed that the impact of such preferential amplification on metagenome sequencing can be avoided by pooling several independent MDA reactions run on a single sample of template environmental DNA [12-17]. However, to our knowledge, the assumption that pooling MDA reactions minimizes representational bias in shotgun metagenome sequence libraries has not been thoroughly tested.

We constructed two mock viral communities to examine the representational bias of MDA treatments versus an unamplified control sample using circular consensus reads from Single Molecule Real-Time (SMRT) sequencing (Pacific Biosciences (PacBio), Menlo Park, CA, USA). SMRT sequencing was ideally suited to the experiment as DNA amplification is not required in the process of preparing DNA fragment libraries for sequencing, whereas Illumina and 454 pyrosequencing technologies employ bridge amplification and emulsion PCR, respectively.

## Methods

### Mock community construction

Two mock bacteriophage communities were constructed. These communities were ideally suited to the experiment as the small genome size of phages enabled us to obtain deep sequence coverage with modest levels of sequencing (one PacBio SMRT cell per community treatment). DNA integrity was assessed by running ≥25 ng DNA on a 0.6% agarose gel. Genomic samples with observed degradation products (T4, VBP32 and VBpm10) were purified using gel extraction to isolate large fragments (>48.5 kb) away from smaller DNA fragments. Phage DNA was quantified using the Qubit Quant-iT dsDNA high-sensitivity kit (Invitrogen, Carlsbad, CA, USA) to calculate the amount of DNA to add for each phage during mock community preparation. The first community comprised of nine mycobacteriophage genomes with a similar %GC content of about 63% GC. Genome populations (phage gDNA) occurred at different frequencies in a tiered structure so that the most abundant and least abundant comprised 28.19% and 0.04% of the community, respectively. The second community included eight phage gDNA samples added at equal-genome equivalents and having a range of %GC content from 35.3 to 67.5%. (Additional file 1: Table S1).

### Amplification treatments

Three library treatment preparations were performed for each community: an unamplified control, a library constructed from a single MDA treatment (MDA1), and a library constructed from a pool of five replicate MDA reactions (MDA5). For the MDA treatments, six reactions per mock community type (tiered and even) were amplified using the Illustra Genomiphi V2 DNA Amplification kit (GE Healthcare, Pittsburgh, PA, USA). Ten nanograms of gDNA per reaction were amplified according to the manufacturer’s instructions. One MDA treatment for each library was run for 2 hours at 30°C and sequenced individually (MDA1 treatment) while five replicate reactions were run for 1.5 hours at 30°C and then pooled together before library preparation and sequencing (MDA5 treatment). No amplification prior to fragment library construction was performed for the control treatment.

### Library preparation and sequencing

One microgram of each DNA treatment (MDA1, MDA5 and control) was prepared for PacBio circular consensus sequencing (CCS) using the 2-kb Template Preparation and Sequencing protocol from Pacific Biosciences. CCS involves the creation of short fragment libraries (500 to 2000 bp) where individual reads are sequenced in multiple passes due to circularization of template molecules using SMRTbell adapters. This allows for the generation of consensus sequences that are higher quality (up to >99% accuracy) than single pass sequences. DNA was fragmented to a target length of 2 kb using Covaris S2 Adaptive Focused Acoustic Disruptor (Covaris, Inc., Woburn, MA, USA) and concentrated using 0.6× volume of Agencourt AMPure XP magnetic beads (Beckman Coulter, Pasadena, CA, USA). Fragmented DNA was end-repaired and SMRTbell adapters were ligated to the blunt ends. SMRTbell templates were purified using 0.6× volume AMPure beads before annealing of the sequencing primer and DNA polymerase. SMRT sequencing was performed at the University of Delaware Sequencing and Genotyping Center using C2/C2 chemistry on a Pacific Biosciences RS sequencer. A total of six samples, consisting of a control, pooled MDA and single MDA sample for each library, were sequenced on separate SMRT cells with 2 × 45 minute movies.

### Analysis of control and multiple displacement amplification treatments

Sequence coverage across each phage genome was assessed to examine the potential impact of MDA amplification on the representation of genomic regions of phage within the mock communities. CCS reads greater than 300 bp from each library were recruited to genome reference sequences using CLC Genomics Workbench version 5.5.1 (Cambridge, MA, USA) using the following mapping parameters: mismatch cost 2, insertion cost 3, deletion cost 3, length fraction 0.5, and similarity fraction 0.8. Sequences used in this recruitment experiment are available through NCBI BioProject PRJNA231204. Mapping at lower stringency allowed chimeric reads in the MDA treatment libraries to recruit to their respective reference genomes, with chimeric regions trimmed out before coverage analyses. Unmapped reads were either host genomic contamination (as determined by BLAST analysis) or poorer quality reads. Since longer reads tend to have higher error scores due to fewer sequencing passes, average read length tended to be higher for the unmapped fraction compared to mapped reads. Results of the CCS recruitment for each community are summarized in Additional file 1: Table S2. Read recruitment was also performed at a similarity fraction of 0.95 and length fractions of 0.6 and 0.9, as two of the genomes in Community 1 (Fruitloop and Wee), were similar, with 94.8% similarity over the first 33.1 kb of their genomes. Nevertheless, the resulting genome coverage pattern for phages Fruitloop and Wee remained the same regardless of the similarity and length settings (Additional file 1: Figure S1). Genome coverage at every position in the reference genome for each treatment was calculated using the mpileup function of SAMtools [18] and graphed using R (version 2.14.0) [19]. Gene coverage for each genome was computed using a custom perl script (Calculation ORF Coverage, http://sourceforge.net/projects/calculationorfcoverage/). Comparison of gene coverage between treatments by performing pairwise t-tests and Pearson’s correlation coefficient was computed using JMP statistical software (version 9.0.0; SAS, Cary, NC, USA).

## Results

The PacBio sequencing technology is particularly sensitive to DNA quality as input DNA is sequenced directly with no prior PCR amplification or cloning steps [20]. The performance of MDA is also dependent on input DNA quality. In a heterogenous mixture of DNA, degraded gDNA will have fewer amplification branches during MDA leading to unbalanced amplification of viral community members [21-23]. Since mock communities were constructed from phage gDNA isolated by multiple laboratories using different DNA extraction techniques and storage conditions, the DNA quality of each viral genome in the mock community was variable. Six of the 15 phage genomes were covered poorly. In the case of the tiered community (Community 1), phages Catera, Angelica and Solon had low coverage because they were designed to be rare members within the mock community. Other phages (T4, VBpm10 and Athena) were poorly covered due to either unknown issues in the sequencing pipeline or possibly poor quality of input phage gDNA. In control mock communities, phages T4, VBpm10 and Athena had lower coverage than expected, likely due to poor DNA quality. Removal of smaller degradation products was attempted for T4 and VBpm10 using gel extraction, but this was likely unsuccessful. Because these three genomes sequenced poorly, the resulting rank genome distribution of phages within the metagenome library did not match the predicted mock community structure. However, the majority of phage genomes in the experiments (five genomes from each community) had sufficient sequencing coverage, and thus it was possible to examine the potential influence of MDA on representation of phage genomic regions (Additional file 1: Table S1).

**Figure 1:**
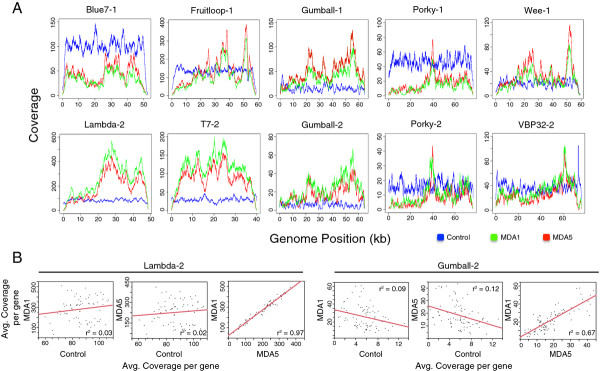
**Sequence coverage of mock viral community genomes from control and multiple displacement amplification treatments. (A)** Depth of coverage across the length of the genome for community members from control and multiple displacement amplification (MDA) treatments. The blue plot represents genome coverage for the control community, the green plot represents genome coverage for the single MDA treatment (MDA1), and the red plot represents genome coverage for the pooled MDA treatment (MDA5). −1 and −2 indicates mock community 1 and mock community 2, respectively. **(B)** Linear regression of pairwise comparison of gene coverage between control, MDA1 and MDA5 treatments for Lambda-2 and Gumball-2. Each point represents a single gene.

Coverage patterns across each genome in both the pooled and single MDA treatments displayed a striking similarity to one another, and differed from the control treatments that tended to have relatively even coverage across the genomes (Figure 1A). In most cases, the coverage plots for the MDA1 and MDA5 treatments were highly similar. In agreement with this observation, genomes from the MDA treated libraries had a greater standard deviation of coverage as compared with genomes in the control treatment (Table 1). This was particularly evident for phage Fruitloop. While average coverage of the Fruitloop genome was similar across treatments, the standard deviation was roughly three times greater in MDA treatments compared to control. Pairwise comparison of average sequence coverage per gene in the treatments indicated a high correlation between MDA treatments (*P* < 0.0001) but not between the MDA treatments and the control. The r^2^ values of the linear regressions ranged from 0.67 to 0.97 (correlation coefficient values of 0.79 to 0.99) in comparisons of average sequence coverage per gene in the MDA1 and MDA5 treatments (Figure 1B, Table 2). Similar comparisons for the control versus MDA1 treatments or control versus MDA5 treatments yielded r^2^ ranges of 0.01 to 0.17 and 0.001 to 0.31, respectively. Interestingly, mycobacteriophages Gumball and Porky, included in both mock communities, had similar gene coverage patterns when compared across treatments (Figure 1A, Table 2) and across communities (Table 3). This suggests that the composition of the mock community did not influence resulting genome coverage patterns, and that MDA biases were likely sequence-dependent.

**Table 1:** Pacific Biosciences circular consensus recruiting to each genome and genome coverage

			**Control**			**MDA5**			**MDA1**		
Genome*	**%GC**	**Predicted read abundance**^**† **^**(%)**	**CCS reads recruited**	**Read abundance (%)**	**Coverage (±SD)**	**CCS reads recruited**	**Read abundance (%)**	**Coverage (±SD)**	**CCS reads recruited**	**Read abundance (%)**	**Coverage (±SD)**
Blue7-1	61.4	15.5	4,631	25.9	98.8 (19.5)	2,380	13.2	43.8 (19.4)	1,522	13.4	33.9 (13.9)
Fuitloop-1	61.8	31.1	7,165	40.1	132.1 (25.5)	8,341	46.4	140.5 (82.4)	5,419	47.8	111.4 (65.7)
Gumball-1	59.6	20.7	1,230	6.9	15.4 (6.1)	3,460	19.2	52.5 (25.1)	2,007	17.7	37.2 (17.9)
Porky-1	63.5	25.9	3,271	18.3	46.3 (7.3)	1,401	7.8	18.1 (12.2)	889	7.8	13.6 (8.8)
Wee-1	61.8	5.2	1,127	6.3	20.3 (5.6)	2,216	12.3	35.8 (22.1)	1,391	12.3	27.3 (15.3)
Gumball-2	59.6	20.8	495	5.4	6.2 (3.0)	1,261	6.5	18.1 (9.4)	1,613	6.5	24.0 (12.6)
Lambda-2	49.9	15.6	3,737	40.7	84.7 (12.0)	10,995	56.3	208.7 (107.1)	14,284	57.5	274.6 (130.7)
Porky-2	63.5	24.5	1,121	12.2	16.1 (3.7)	664	3.4	8.2 (6.5)	815	3.3	10.1 (7.3)
T7-2	48.4	12.8	1,050	11.4	29.8 (5.6)	3,920	20.1	90.2 (30.7)	5,029	20.2	115.7 (37.7)
VBP32-2	42.5	24.9	2,616	28.5	37.5 (8.9)	2,373	12.1	27.6 (15.9)	2,821	11.4	33.5 (17.2)

*-1, -2 indicates data from community one and community two, respectively. ^†^Predicted read abundances were recalculated to take into account the low recruitment of phage Athena, T4 and VBpm10. CCS, circular consensus sequencing; MDA, multiple displacement amplification.

**Table 2:** Correlation coefficient of pairwise comparison of gene coverage in control and multiple displacement amplification treatments

	**Pearson’s correlation coefficient**		
	**Treatments**	**Control**	**Single MDA**
**Blue7**	Single MDA	0.21^†^	
	Pooled MDA	0.37^†^	0.86^‡^
**Fruitloop**	Single MDA	0.07	
	Pooled MDA	0.04	0.98^‡^
**Gumball-1**	Single MDA	−0.31^†^	
	Pooled MDA	−0.33^†^	0.94^‡^
**Gumball-2**	Single MDA	−0.31^†^	
	Pooled MDA	−0.36^†^	0.82^‡^
**Lambda**	Single MDA	0.16	
	Pooled MDA	0.10	0.99^‡^
**Porky-1**	Single MDA	0.18^†^	
	Pooled MDA	0.15	0.91^‡^
**Porky-2**	Single MDA	−0.15	
	Pooled MDA	−0.09	0.79^‡^
**T7**	Single MDA	−0.42^†^	
	Pooled MDA	−0.56^†^	0.95^‡^
**VBP32**	Single MDA	−0.11	
	Pooled MDA	−0.15	0.92^‡^
**Wee**	Single MDA	0.24^†^	
	Pooled MDA	0.22^†^	0.93^‡^

Comparisons were of average coverage for each predicted gene in a genome. ^†^*P* < 0.05.

^‡^*P* < 0.0001. MDA, multiple displacement amplification.

**Table 3:** Correlation coefficient of pairwise comparison of gene coverage across communities for mycobacteriophage Gumball and Porky

	**Pearson’s correlation coefficient**		
		**Gumball-2**	
	**Treatments**	**Single MDA**	**Pooled MDA**
**Gumball-1**	Single MDA	0.92^‡^	0.88^‡^
	Pooled MDA	0.90^‡^	0.89^‡^
		**Porky-2**	
	**Treatments**	**Single MDA**	**Pooled MDA**
**Porky-1**	Single MDA	0.86^‡^	0.85^‡^
	Pooled MDA	0.84^‡^	0.88^‡^

Comparisons were of average coverage for each predicted gene in a genome.

^‡^*P* < 0.0001. MDA, multiple displacement amplification.

Coverage bias in the MDA treatments occurred towards the middle of the genome for several phages (Blue7, Porky, Wee, lambda, Fruitloop, T7, and Gumball) relative to the ends of the genome (Figure 1A). The bias towards the middle is understandable as MDA priming events producing fragments of sufficient length for sequencing would likely have proceeded towards the middle of the linear genome thus leading to an over-representation of DNA (and subsequently sequence reads) in the middle of the phage genome. A few genomes also showed coverage peaks within 10 kb of one or both ends (lambda, Blue7, VBP32, Wee, Gumball, and Fruitloop). These peaks are difficult to explain, but may have resulted from a bias in the priming efficiency of subsets of the random hexamers used in priming the MDA reaction [24,25]. Five to 1,140 bp were missing from genome termini in both MDA treatments, with the notable exception of Gumball and VBP32 which have terminally redundant genomes. This phenomena of missing bases at the ends of linear genomes has been reported before in the sequencing of chromosomal ends [22,26,27] and is likely the result of DNA fragments becoming progressively shorter as priming events near the terminal end of a genome. Subsequently these short fragments are lost during library construction or filtered out in bioinformatic processing and longer fragments containing the ends are rare within the sequence library.

## Discussion

An important aim of metagenomics is to assess the frequency of taxa and gene functions within natural microbial communities through DNA sequence data. The rigor of these assessments rests on how well the frequency of a sequence within a metagenome library reflects the frequency of its originating microbial population within the community. These data indicate that the frequency of sequence reads from a viral community gDNA sample amplified using MDA does not accurately reflect the true frequency of taxa or gene functions among viral populations within the original sample. MDA clearly caused certain regions of the phage genomes to be over-represented in the resulting sequence library. Counter to current thinking, pooling of several MDA reactions did not alleviate this bias as coverage patterns within genomes were recurrent across experiments and reactions. The most parsimonious explanation for this phenomenon is that the random hexamers used for priming the MDA reaction did not in fact prime randomly across all genomes. The consequence of unequal priming efficiency of MDA was that subsets of genes from a given viral genome were artificially over- or under-represented within the resulting metagenome sequence library.

Many viral genomes, especially phage genomes, have a modular genetic organization with genes clustered according to their functional roles such as head assembly, tail assembly and genome replication [28]. Because the middle portions of linear phage genomes tended to be over-represented, genes within these regions would also be over-represented within the library relative to their true abundance within the genomes. Many phages have similar functions located at similar locations in their genomes, such as the λ supergroup within the siphoviridae family [29]. At the community scale, inaccuracies in the frequency of gene functional groups caused by MDA could be linked with the typical position of a given functional gene group within a phage genome. It should also be noted that non-uniform coverage could hamper assembly-based community analyses that strive to assemble genome-length fragments from a complex mixture of multiple genotypes [30,31].

Considerable effort has been focused on evaluating and optimizing methods for metagenomic library construction. LASL is a commonly utilized alternative to MDA for preparing metagenomic libraries [1,4,32,33]. While starting DNA quantities as low as 1 pg have been successfully prepared for Illumina sequencing using the LASL, such low starting amounts of DNA require more PCR cycles to generate sufficient DNA for sequencing. As a consequence, sequences at the extremes of %GC content can be under-represented. At greater initial DNA quantities (10 to 100 ng), fewer PCR cycles are needed leading to a smaller degree of %GC bias [1]. Initial analyses of a relatively new technique, known as LADS, indicate that LADS libraries produced more uniform coverage than PCR-based library preparations across low and high %GC genome regions [3]. However, the LADS procedure has been found to generate a greater number of duplicate and chimeric reads as compared to standard Illumina library protocols [34]. More research is needed to evaluate the performance of LADS for metagenomic investigations. Transposase-based Nextera™ kits have been increasingly utilized in the construction of metagenomic fragment libraries for Illumina sequencing. While better suited to high-throughput sample preparation, Nextera also suffers from %GC biases linked to the PCR step and a slight bias in sequence targeting by the transposase during DNA fragmentation [2,4,35]. Despite the documented biases of the LASL and Nextera protocols, the degree of bias in these techniques is substantially lower than that of MDA protocols [9,33,36].

In theory, any amount of amplification has the potential to skew the ambient distribution of mixed community DNA. Therefore, an optimal library preparation would require no amplification steps. PCR-free protocols are available, but the large amount of input DNA needed for such procedures can be prohibitive for ecological studies [37]. The advent of new sequencing technologies coupled with new protocols to prepare DNA for sequencing are paving the way for future methodologies that may exclude any type of amplification. Library preparation methods that require as little as 1 ng DNA have been demonstrated for PacBio SMRT sequencing [38]. With continuing development, such methodologies hold promise for removing amplification bias from metagenomic investigations.

## Conclusions

Our findings contribute to the growing evidence that MDA should not be utilized in metagenomic studies seeking quantitative information on the population structure of a microbial community. MDA has been an invaluable tool in several important areas of research, including single cell genomics and forensics [7,32,33,39]. The efficient amplification of circular ssDNA templates during MDA has been exploited to explore the diversity of ssDNA viruses [40-43]. Within microbiome research, MDA protocols are an easy means of obtaining sufficient DNA for next generation sequencing; however, subsequent observations of microbial taxa and gene functions within metagenome libraries are not quantitative. The practice of pooling replicate MDA reactions from a single sample does not alleviate biases in the representation of sequences within a library. Researchers should carefully evaluate their requirements for quantitative data on the frequency of microbial taxa and gene functions before utilizing MDA in a microbiome investigation.

## Abbreviations

bp: base pair; CCS: circular consensus sequencing; gDNA: genomic DNA; LADS: linear amplification for deep sequencing; LASL: linker amplified shotgun library; MDA: multiple displacement amplification; PacBio: Pacific Biosciences; PCR: polymerase chain reaction; SMRT: Single Molecule Real-Time; ssDNA: single stranded DNA.

## Competing interests

The authors declare that they have no competing interests.

## Authors’ contributions

RM carried out the design and constructed the mock viral communities, analyzed sequencing data, performed statistical analyses and drafted the manuscript. CM and VV participated in construction of the mock viral communities and sequencing analysis. DN and EC participated in the bioinformatic analyses. SWP and KEW were involved in the design of the experiment and the drafting of the manuscript. All authors read and approved the final manuscript.

## Supplementary Material

Additional file 1 **Table S1.** Bacteriophage genomes within two mock viral communities. **Table S2.** Results of Pacific Biosciences circular consensus sequencing read recruitment to reference genomes. **Figure S1.** Coverage patterns of Fruitloop and Wee for control and multiple displacement amplification treatments using A) 95% similarity and 60% length fraction and B) 95% similarity and 90% length fraction for reference mapping parameters. Click here for file

## References

[B1] Duhaime MB Deng L Poulos BT Sullivan MB Towards quantitative metagenomics of wild viruses and other ultra-low concentration DNA samples: a rigorous assessment and optimization of the linker amplification method Environ Microbiol 2012 14 2526 2537 10.1111/j.1462-2920.2012.02791.x 22713159 PMC3466414

[B2] Marine R Polson SW Ravel J Hatfull G Russell D Sullivan M Syed F Dumas M Wommack KE Evaluation of a transposase protocol for rapid generation of shotgun high-throughput sequencing libraries from nanogram quantities of DNA Appl Environ Microbiol 2011 77 8071 8079 10.1128/AEM.05610-11 21948828 PMC3209006

[B3] Hoeijmakers WAM Bártfai R Françoijs K Stunnenberg HG Linear amplification for deep sequencing Nat Protoc 2011 6 1026 1036 10.1038/nprot.2011.345 21720315

[B4] Solonenko SA Ignacio-Espinoza JC Alberti A Cruaud C Hallam S Konstantinidis K Tyson G Wincker P Sullivan MB Sequencing platform and library preparation choices impact viral metagenomes BMC Genomics 2013 14 320 10.1186/1471-2164-14-320 23663384 PMC3655917

[B5] Thurber RV Haynes M Breitbart M Wegley L Rohwer F Laboratory procedures to generate viral metagenomes Nat Protoc 2009 4 470 483 10.1038/nprot.2009.10 19300441

[B6] Lasken RS Egholm M Whole genome amplification: abundant supplies of DNA from precious samples or clinical specimens Trends Biotechnol 2003 21 531 535 10.1016/j.tibtech.2003.09.010 14624861

[B7] Binga EK Lasken RS Neufeld JD Something from (almost) nothing: the impact of multiple displacement amplification on microbial ecology ISME J 2008 2 233 241 10.1038/ismej.2008.10 18256705

[B8] Polson SW Wilhelm SW Wommack KE Unraveling the viral tapestry (from inside the capsid out) ISME J 2011 5 165 168 10.1038/ismej.2010.81 20555364 PMC3105704

[B9] Yilmaz S Allgaier M Hugenholtz P Multiple displacement amplification compromises quantitative analysis of metagenomes Nat Methods 2010 7 943 944 10.1038/nmeth1210-943 21116242

[B10] Dichosa AEK Fitzsimons MS Lo C Weston LL Preteska LG Snook JP Zhang X Gu W McMurry K Green LD Chain PS Detter JC Han CS Artificial polyploidy improves bacterial single cell genome recovery PLoS One 2012 7 e37387 10.1371/journal.pone.0037387 22666352 PMC3359284

[B11] Wang J Van Nostrand JD Wu L He Z Li G Zhou J Microarray-based evaluation of whole-community genome DNA amplification methods Appl Environ Microbiol 2011 77 4241 4245 10.1128/AEM.01834-10 21498751 PMC3131669

[B12] Abulencia CB Wyborski DL Garcia JA Podar M Chen W Chang SH Chang HW Watson D Brodie EL Hazen TC Keller M Environmental whole-genome amplification to access microbial populations in contaminated sediments Appl Environ Microbiol 2006 72 3291 3301 10.1128/AEM.72.5.3291-3301.2006 16672469 PMC1472342

[B13] Dinsdale EA Edwards RA Hall D Angly F Breitbart M Brulc JM Furlan M Desnues C Haynes M Li L McDaniel L Moran MA Nelson KE Nilsson C Olson R Paul J Brito BR Ruan Y Swan BK Stevens R Valentine DL Thurber RV Wegley L White BA Rohwer F Functional metagenomic profiling of nine biomes Nature 2008 452 629 632 10.1038/nature06810 18337718

[B14] Dinsdale EA Pantos O Smriga S Edwards RA Microbial ecology of four coral atolls in the Northern Line Islands PLoS One 2008 3 e1584 10.1371/journal.pone.0001584 18301735 PMC2253183

[B15] Cassman N Prieto-Davó A Walsh K Silva GGZ Angly F Akhter S Barott K Busch J McDole T Haggerty JM Willner D Alarcón G Ulloa O DeLong EF Dutilh BE Rohwer F Dinsdale EA Oxygen minimum zones harbour novel viral communities with low diversity Environ Microbiol 2012 14 3043 3065 10.1111/j.1462-2920.2012.02891.x 23039259

[B16] Hewson I Barbosa JG Brown JM Donelan RP Eaglesham JB Eggleston EM Labarre BA Temporal dynamics and decay of putatively allochthonous and autochthonous viral genotypes in contrasting freshwater lakes Appl Environ Microbiol 2012 78 6583 6591 10.1128/AEM.01705-12 22773646 PMC3426699

[B17] Willner D Furlan M Haynes M Schmieder R Angly FE Silva J Tammadoni S Nosrat B Conrad D Rohwer F Metagenomic analysis of respiratory tract DNA viral communities in cystic fibrosis and non-cystic fibrosis individuals PLoS One 2009 4 e7370 10.1371/journal.pone.0007370 19816605 PMC2756586

[B18] Li H Handsaker B Wysoker A Fennell T Ruan J Homer N Marth G Abecasis G Durbin R 1000 Genome Project Data Processing Subgroup The sequence alignment/Map format and SAMtools Bioinformatics 2009 25 2078 2079 10.1093/bioinformatics/btp352 19505943 PMC2723002

[B19] R Project for Statistical Computing [http://www.r-project.org/]

[B20] Pacific biosciences technical notes, microbial assembly experimental design [http://www.pacificbiosciences.com/pdf/TechnicalNote_Experimental_Design_for_Microbial_Assembly.pdf]

[B21] Bergen AW Effects of electron-beam irradiation on whole genome amplification Cancer Epidem Biomar 2005 14 1016 1019 10.1158/1055-9965.EPI-04-0686 15824182

[B22] Lage JM Leamon JH Pejovic T Hamann S Lacey M Dillon D Segraves R Vossbrinck B González A Pinkel D Albertson DG Costa J Lizardi PM Whole genome analysis of genetic alterations in small DNA samples using hyperbranched strand displacement amplification and array-CGH Genome Res 2003 13 294 307 10.1101/gr.377203 12566408 PMC420367

[B23] Mead S Poulter M Beck J Uphill J Jones C Ang CE Mein CA Collinge J Successful amplification of degraded DNA for use with high-throughput SNP genotyping platforms Hum Mutat 2008 29 1452 1458 10.1002/humu.20782 18551557

[B24] Marcy Y Ishoey T Lasken RS Stockwell TB Walenz BP Halpern AL Beeson KY Goldberg SMD Quake SR Nanoliter reactors improve multiple displacement amplification of genomes from single cells PLoS Genet 2007 3 1702 1708 17892324 10.1371/journal.pgen.0030155 PMC1988849

[B25] Hansen KD Brenner SE Dudoit S Biases in Illumina transcriptome sequencing caused by random hexamer priming Nucleic Acids Res 2010 38 e131 10.1093/nar/gkq224 20395217 PMC2896536

[B26] Panelli S Damiani G Espen L Sgaramella V Ligation overcomes terminal underrepresentation in multiple displacement amplification of linear DNA Biotechniques 2005 39 174 180 10.2144/05392BM03 16116788

[B27] Tzvetkov MV Becker C Kulle B Nürnberg P Brockmöller J Wojnowski L Genome-wide single-nucleotide polymorphism arrays demonstrate high fidelity of multiple displacement-based whole-genome amplification Electrophoresis 2005 26 710 715 10.1002/elps.200410121 15690424

[B28] Krupovic M Prangishvili D Hendrix RW Bamford DH Genomics of bacterial and archaeal viruses: dynamics within the prokaryotic virosphere Microbiol Mol Biol Rev 2011 75 610 635 10.1128/MMBR.00011-11 22126996 PMC3232739

[B29] Brüssow H Desiere F Comparative phage genomics and the evolution of Siphoviridae: insights from dairy phages Mol Microbiol 2001 39 213 222 10.1046/j.1365-2958.2001.02228.x 11136444

[B30] Kunin V Copeland A Lapidus A Mavromatis M Hugenholtz P A bioinformatician's guide to metagenomics Microbiol Mol Biol Rev 2008 72 557 10.1128/MMBR.00009-08 19052320 PMC2593568

[B31] Mavromatis K Ivanova N Barry K Shapiro H Goltsman E McHardy AC Rigoutsos I Salamov A Korzeniewski F Land M Lapidus A Grigoriev I Richardson P Hugenholtz P Kyrpides NC Use of simulated data sets to evaluate the fidelity of metagenomic processing methods Nat Methods 2007 4 495 500 10.1038/nmeth1043 17468765

[B32] Henn MR Sullivan MB Stange-Thomann N Osburne MS Berlin AM Kelly L Yandava C Kodira C Zeng Q Weiand M Sparrow T Saif S Giannoukos G Young SK Nusbaum C Birren BW Chisholm SW Analysis of high-throughput sequencing and annotation strategies for phage genomes PLoS One 2010 5 e9083 10.1371/journal.pone.0009083 20140207 PMC2816706

[B33] Kim KH Bae JW Amplification methods bias metagenomic libraries of uncultured single-stranded and double-stranded DNA viruses Appl Environ Microbiol 2011 77 7663 7668 10.1128/AEM.00289-11 21926223 PMC3209148

[B34] Oyola SO Otto TD Gu Y Maslen G Manske M Campino S Turner DJ Macinnis B Kwiatkowski DP Swerdlow HP Quail MA Optimizing Illumina next-generation sequencing library preparation for extremely AT-biased genomes BMC Genomics 2012 13 1 10.1186/1471-2164-13-1 22214261 PMC3312816

[B35] Adey A Morrison HG Asan Xun X Kitzman JO Turner EH Stackhouse B MacKenzie AP Caruccio NC Zhang X Shendure J Rapid, low-input, low-bias construction of shotgun fragment libraries by high-density in vitro transposition Genome Biol 2010 11 R119 10.1186/gb-2010-11-12-r119 21143862 PMC3046479

[B36] Pinard R de Winter A Sarkis GJ Gerstein MB Tartaro KR Plant RN Egholm M Rothberg JM Leamon JH Assessment of whole genome amplification-induced bias through high-throughput, massively parallel whole genome sequencing BMC Genomics 2006 7 216 10.1186/1471-2164-7-216 16928277 PMC1560136

[B37] Kozarewa I Ning Z Quail MA Sanders MJ Berriman M Turner DJ Amplification-free Illumina sequencing-library preparation facilitates improved mapping and assembly of (G + C)-biased genomes Nat Methods 2009 6 291 295 10.1038/nmeth.1311 19287394 PMC2664327

[B38] Coupland P Chandra T Quail M Reik W Swerdlow H Direct sequencing of small genomes on the pacific biosciences RS without library preparation Biotechniques 2012 53 365 372 23227987 10.2144/000113962 PMC3808810

[B39] Raghunathan A Ferguson HR Bornarth CJ Song W Driscoll M Lasken RS Genomic DNA amplification from a single bacterium Appl Environ Microbiol 2005 71 3342 3346 10.1128/AEM.71.6.3342-3347.2005 15933038 PMC1151817

[B40] Desnues C Rodriguez-Brito B Rayhawk S Kelley S Tran T Haynes M Liu H Furlan M Wegley L Chau B Ruan Y Hall D Angly FE Edwards RA Li L Thurber RV Reid RP Siefert J Souza V Valentine DL Swan BK Breitbart M Rohwer F Biodiversity and biogeography of phages in modern stromatolites and thrombolites Nature 2008 452 340 343 10.1038/nature06735 18311127

[B41] Kim KH Chang HW Nam YD Roh SW Kim MS Sung Y Jeon CO Oh HM Bae JW Amplification of uncultured single-stranded DNA viruses from rice paddy soil Appl Environ Microbiol 2008 74 5975 5985 10.1128/AEM.01275-08 18708511 PMC2565953

[B42] Kim M Park E Roh SW Bae J Diversity and abundance of single-stranded DNA viruses in human feces Appl Environ Microbiol 2011 77 8062 8070 10.1128/AEM.06331-11 21948823 PMC3208976

[B43] Rosario K Nilsson C Lim YW Ruan Y Breitbart M Metagenomic analysis of viruses in reclaimed water Environ Microbiol 2009 11 2806 2820 10.1111/j.1462-2920.2009.01964.x 19555373

