# Supplementary material for: Caught in the middle with multiple displacement amplification: the myth of pooling for avoiding multiple displacement amplification bias in a metagenome
Source: Microbiome. 2014 Jan 30;2:3. doi: 10.1186/2049-2618-2-3 (PMC3937105; doi:10.1186/2049-2618-2-3)

Supplementary Table 1. Bacteriophage genomes within two mock viral communities.

Community 1					Community 2				
**NCBI/GenBank Accession #**	**Genomes**	**GC (%)**	**Genome Length (bp)**	**Predicted Read Abundance (%)**	**NCBI/GenBank Accession #**	**Genomes**	**GC (%)**	**Genome Length (bb)**	**Predicted Read Abundance (%)**
NC_011288	Fruitloop	61.8	58,471	28.2	NC_000866	T4*	35.3	168,903	29.4
NC_011055	Porky	63.5	76,312	23.5	PRJNA47675	VBP32	42.5	76,212	5.6
NC_011290	Gumball	59.6	64,807	18.8	PRJNA47683	VBpm10*	46.1	32,225	13.2
JN698999	Blue7	61.4	52,288	14.1	NC_001604	T7	48.4	39,937	8.4
JN699003	Athena*	67.5	69,409	9.4	NC_001416	Lambda	49.9	48,503	7.0
NC_014901	Wee	61.8	59,230	4.7	NC_011290	Gumball	59.6	64,807	12.0
NC_008207	Catera*	64.7	153,766	1.2	NC_011055	Porky	63.5	76,312	13.2
NC_014458	Angelica*	66.4	59,598	0.12	JN699003	Athena*	67.5	69,409	11.2
NC_011267	Solon*	63.8	49,487	0.04					

*Not included in analyses due to low sequence coverage

Supplementary Table 2. Results of Pacific Biosciences circular consensus sequencing read recruitment to reference genomes

Community	Number of CCS reads	Mapped CCS reads	Percentage of CCS reads mapped	Average Read Length of CCS reads (±S.D.)		Max. Read Length (bp)	Mismatch Rate per 100 aligned bases*	Average Indel Rate per 100 aligned bases*
				Mapped Reads	Unmapped Reads			
**Control-1**	17,957	17,854	99.4	1066 (359)	1102 (444)	2600	0.4	1.8
**Pooled-1**	18,647	17,984	96.4	957 (343)	1172 (399)	2552	3.0	1.5
**Single-1**	11,944	11,346	95.0	1175 (345)	1281 (347)	2637	3.3	1.6
**Control-2**	9,261	9,179	99.1	1077 (361)	1031 (413)	2504	0.2	1.7
**Pooled-2**	20,589	19,539	94.9	911 (335)	1098 (372)	2466	4.0	1.6
**Single-2**	26,147	24,849	95.0	926 (359)	1149 (431)	2654	4.0	1.5

*The rate of mismatched bases and indels (insertions/deletions) per 100 bp was calculated for reads recruited to genomes with sufficient coverage from each community (Table S1) using Picard Tools (http://picard.sourceforge.net). The higher mismatch rate for the Pooled and Single treatments reflects mismatches due to chimeric reads. After trimming of chimeric regions, the rate of mismatches for the MDA treatment libraries was similar to control libraries (data not shown).

Supplementary Figure S1. Coverage patterns of Fruitloop and Wee for control and multiple displacement amplification treatments using A) 95% similarity and 60% length fraction and B) 95% similarity and 90% length fraction for reference mapping

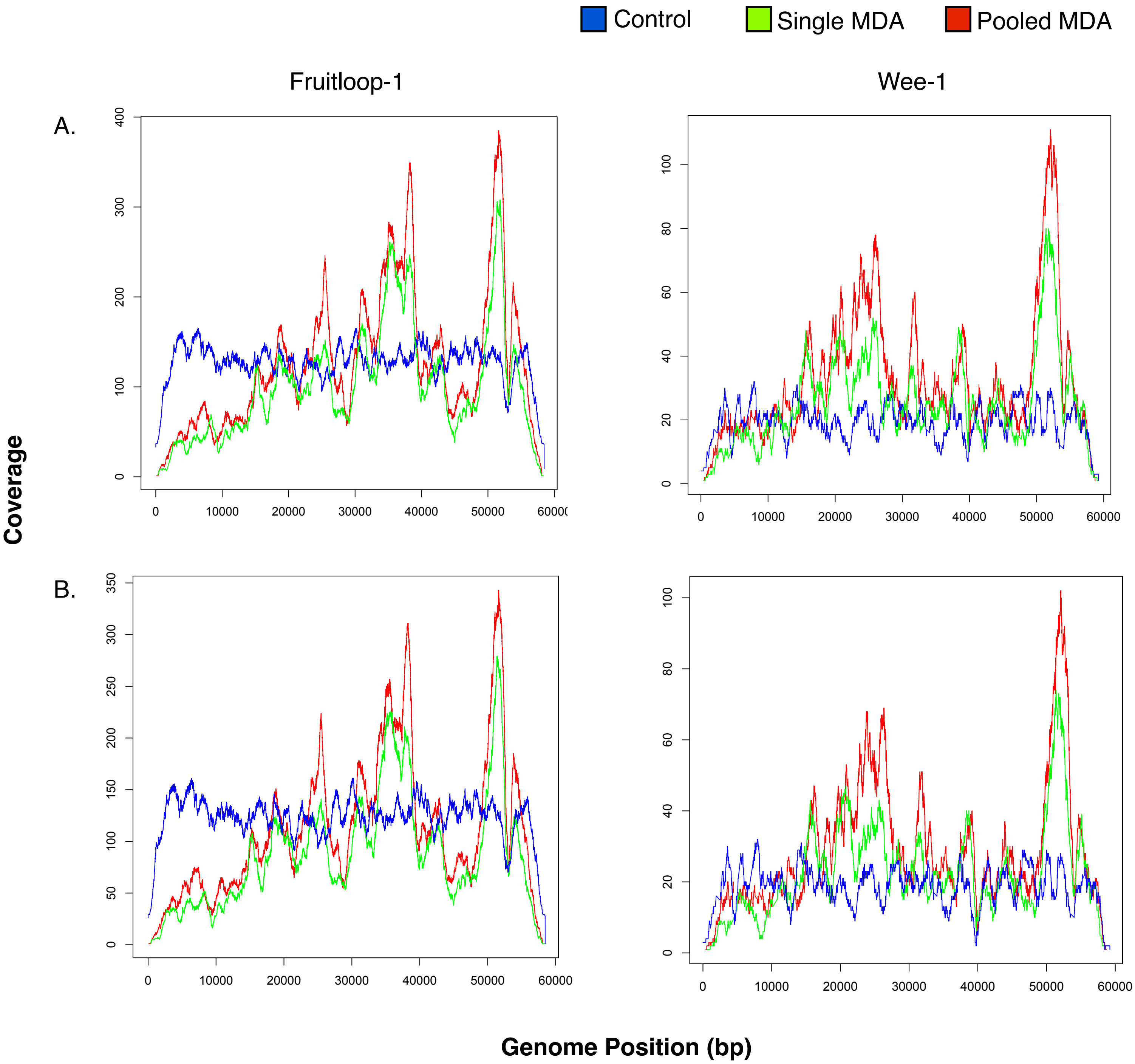

Supplement: Additional file 1 — Table S1. Bacteriophage genomes within two mock viral communities. Table S2. Results of Pacific Biosciences circular consensus sequencing read recruitment to reference genomes. Figure S1. Coverage patterns of Fruitloop and Wee for control and multiple displacement amplification treatments using A) 95% similarity and 60% length fraction and B) 95% similarity and 90% length fraction for reference mapping parameters. [file 2049-2618-2-3-S1.pdf]
